# Attribute Reduction Based on Property Pictorial Diagram

**DOI:** 10.1155/2014/109706

**Published:** 2014-08-27

**Authors:** Qing Wan, Ling Wei

**Affiliations:** School of Mathematics, Northwest University, Xi'an, Shaanxi 710069, China

## Abstract

This paper mainly studies attribute reduction which keeps the lattice structure in formal contexts based on the property pictorial diagram. Firstly, the property pictorial diagram of a formal context is defined. Based on such diagram, an attribute reduction approach of concept lattice is achieved. Then, through the relation between an original formal context and its complementary context, an attribute reduct of complementary context concept lattice is obtained, which is also based on the property pictorial diagram of the original formal context. Finally, attribute reducts in property oriented concept lattice and object oriented concept lattice can be acquired by the relations of attribute reduction between these two lattices and concept lattice of complementary context. In addition, a detailed illustrative example is presented.

## 1. Introduction

Formal concept analysis (FCA) [1, 2], introduced by German mathematician Wille in 1982, has become one of the important tools for knowledge discovery and data analysis. The basic notions of FCA are formal context, formal concept, and the corresponding concept lattice. Another useful tool for knowledge discovery and data analysis is rough set theory (RST), originally proposed by Pawlak in 1982 [3, 4], in which the lower and upper approximations of an arbitrary subset of universe are the basics. At present, FCA and RST have many important applications in various fields, respectively, and many efforts have been made to compare and combine them. For example, Düntsch and Gediga [5] introduced the notions of rough set theory into formal concept analysis and proposed property oriented concept lattice. Based on such idea, Yao [6] proposed object oriented concept lattice. Shi et al. [7] and Wang and Zhang [8] studied the relation between RST and FCA. Then, Wei and Qi [9] discussed the relation between concept lattice reduction and rough set reduction. Liu et al. [10] studied the reduction of the concept lattices based on rough set theory. Wang [11] defined “the notions of attribute reduction in concept lattices in a similar way with that in rough set theory.” Poelmans et al. [12] gave “a systematic overview of the more than 120 papers published between 2003 and 2011 on FCA with fuzzy attributes and rough FCA.” Their research enriched the FCA and RST.

Attribute reduction is one of the key issues of RST and FCA. In the case of RST, attribute reduction in information systems is based on equivalence relation. A reduct is a minimum subset of attributes that provides the same classification ability as the entire set of attributes [3]. Skowron and Rauszer [13] proposed a reduct construction method based on the discernibility matrix, and many researchers improved this method [14–16].

While, there are some differences between the reduction in FCA and in RST. In the case of FCA, Ganter and Wille [1] proposed the term of reduction from the viewpoint of deleting rows or columns. In [17], Zhang et al. presented attribute reduction approaches to finding minimal attribute sets which can determine all extents and their original hierarchy in a formal context. That is to say, the reduction theory can keep the lattice structure of a formal context. Based on such reduction, the authors also studied the reduction theory of formal decision contexts [18]. This approach to attribute reduction is based on the discernibility matrix in FCA. For object oriented concept lattice and property oriented concept lattice, the reduction of keeping their lattice structure was studied by Liu and Wei [19].

Besides this kind of reduction that keeps the lattice structure, there are other reduction theories about formal contexts. For example, Wang and Ma [20] proposed another approach to attribute reduction that can only preserve the extents of meet-irreducible elements in the original concept lattice and described attribute characteristics using meet-irreducible elements. For the object oriented concept lattice and property oriented concept lattice, Wang and Zhang [21] further studied such reduction and described attribute characteristics using meet- (join-) irreducible elements. Medina [22] obtained the relation of attribute reduction among complementary context concept lattice, object oriented concept lattice, and property oriented concept lattice. Wu et al. [23] proposed the granular reduction from the viewpoint of keeping object concepts and discussed information granules and their properties in a formal context. Li et al. [24] constructed “a new framework of knowledge reduction in which the capacity of one concept lattice implying another is defined to measure the significance of the attributes in a consistent decision formal context.” Shao et al. [25] formulated “an approach to attribute reduction in formal decision contexts such that rules extracted from the reduced formal decision contexts are identical to that extracted from the initial formal decision contexts.” Aswani Kumar and Srinivas [26] proposed “a new method based on fuzzy K-means clustering for reducing the size of the concept lattices.”

In this paper, based on [20, 22], we discuss the reduction of a formal context which can keep the lattice structure using arrow relation ↗ defined by Ganter and Wille [1]. First of all, we obtain the new approach to acquiring the arrow relation ↗ on the basis of a property pictorial diagram defined by us. Then, combining the relations between arrow relation ↗, meet-irreducible elements, and attribute characteristics, we present an approach to construct attribute reducts of concept lattice, complementary context concept lattice, object oriented concept lattice, and property oriented concept lattice.

The rest of the paper is organized as follows.  Section 2 reviews the basic notions of FCA.  Section 3 constructs attribute reducts based on property pictorial diagram of a formal context.  Section 4 uses an UCI database to explain our approach in more detail.  Section 5 concludes the paper.

## 2. Preliminaries

In this section, we recall some basic notions in formal concept analysis [1, 2].

**Definition 1 (see [1]).**

A formal context (*G*, *M*, *I*) consists of two sets *G* and *M* and a relation *I* between *G* and *M*. The elements of *G* are called the objects and the elements of *M* are called the attributes of the context. In order to express that an object *g* is in a relation *I* with an attribute *m*, we write *gIm* or (*g*, *m*) ∈ *I* and read it as “the object *g* has the attribute *m*.”

Let (*G*, *M*, *I*) be a formal context. For *A*⊆*G*, *B*⊆*M*, two operators are defined as follows:

(1) $$\begin{array}{c} A^{*}=\left\{m\in M\;|\;\left(g,m\right)\in I\;\;\forall g\in A\right\},\\ B^{\prime }=\left\{g\in G\;|\;\left(g,m\right)\in I\;\;\forall m\in B\right\}. \end{array}$$

(*A*, *B*) is called a formal concept, for short a concept, if and only if A* = *B*, *A* = *B*′, where *A* is called the extent of the formal concept and *B* is called its intent. Particularly, (*m*′, *m* ^′∗^) is a formal concept and is called an attribute concept, and *m*′ is called attribute extent [1]. The set of all concepts of (*G*, *M*, *I*) is denoted by *L*(*G*, *M*, *I*). For any (*A*_1_, *B*_1_), (*A*_2_, *B*_2_) ∈ *L*(*G*, *M*, *I*), we have (*A*_1_, *B*_1_)⩽(*A*_2_, *B*_2_)⇔*A*_1_⊆*A*_2_(⇔*B*_1_⊇*B*_2_). And the infimum and supremum are given by

(2) (A1,B1)∧(A2,B2)=(A1∩A2,(B1∪B2)′∗),(A1,B1)∨(A2,B2)=((A1∪A2)∗′,B1∩B2).

Thus, *L*(*G*, *M*, *I*) is a complete lattice and is called the concept lattice.

To simplify, for all *g* ∈ *G*, for all *m* ∈ *M*, {*g*}* and {*m*}′ are replaced by g* and *m*′, respectively. If, for all *g* ∈ *G*, g* ≠ *∅*, g* ≠ *M*, and, for all *m* ∈ *M*, *m*′ ≠ *∅*, *m*′ ≠ *G*, then the formal context is called canonical. That is to say, there is neither full row/column nor empty row/column in a formal context. Noting this, an irregular formal context can be regularized by removing the full row/column and empty row/column. Such way of regularization causes no effect on the analysis results of the formal context. Thus, without loss of generality, we suppose that all formal contexts are finite and canonical in this paper.

Let (*G*, *M*, *I*) be a formal context. Denote *I* ^*c*^ = (*G* × *M*)∖*I*; then we call (*G*, *M*, *I* ^*c*^) the complementary context of (*G*, *M*, *I*) [1]; the mappings defined in (1) on (*G*, *M*, *I* ^*c*^) are denoted by ^∗*c*^ and ^′*c*^.

All concepts of (*G*, *M*, *I* ^*c*^) are denoted by *L*_*C*_(*G*, *M*, *I*), which is also a complete lattice.

Let (*G*, *M*, *I*) be a formal context. For any *A*⊆*G*, *B*⊆*M*, Düntsch and Gediga defined a pair of approximate operators *♢*, □ as follows [5]:

(3) A♢={m∈M ∣ m′∩A≠∅},B♢={g∈Gg∗∩B≠∅},A□={m∈M ∣ m′⊆A},B□={g∈G ∣ g∗⊆B}.

A pair (*A*, *B*), *A*⊆*G*, *B*⊆*M*, is called a property oriented concept if *A* ^*♢*^ = *B* and *B* ^□^ = *A*. All property oriented concepts of (*G*, *M*, *I*) are denoted by *L*_*P*_(*G*, *M*, *I*). For any (*A*_1_, *B*_1_), (*A*_2_, *B*_2_) ∈ *L*_*P*_(*G*, *M*, *I*), (*A*_1_, *B*_1_)⩽(*A*_2_, *B*_2_)⇔*A*_1_⊆*A*_2_(⇔*B*_1_⊆*B*_2_). And the infimum and supremum are given by

(4) (A1,B1)∧(A2,B2)=(A1∩A2,(B1∩B2)□♢),(A1,B1)∨(A2,B2)=((A1∪A2)♢□,B1∪B2).

Thus, *L*_*P*_(*G*, *M*, *I*) is a complete lattice and is called the property oriented concept lattice.

Based on the work of Düntsch and Gediga, Yao proposed the object oriented concept lattice [6].

A pair (*A*, *B*), *A*⊆*G*, *B*⊆*M*, is called an object oriented concept if *A* ^□^ = *B* and *B* ^*♢*^ = *A*. All object oriented concepts of (*G*, *M*, *I*) are denoted by *L*_*O*_(*G*, *M*, *I*). For any (*A*_1_, *B*_1_), (*A*_2_, *B*_2_) ∈ *L*_*O*_(*G*, *M*, *I*), (*A*_1_, *B*_1_)⩽(*A*_2_, *B*_2_)⇔*A*_1_⊆*A*_2_(⇔*B*_1_⊆*B*_2_). And the infimum and supremum are given by

(5) (A1,B1)∧(A2,B2)=((A1∩A2)□♢,B1∩B2),(A1,B1)∨(A2,B2)=(A1∪A2,(B1∪B2)♢□).

Hence, *L*_*O*_(*G*, *M*, *I*) is a complete lattice and is called the object oriented concept lattice [6, 27].

Thus, for one formal context (*G*, *M*, *I*), we have four different lattices, concept lattice *L*(*G*, *M*, *I*), complementary context concept lattice *L*_*C*_(*G*, *M*, *I*), property oriented concept lattice *L*_*P*_(*G*, *M*, *I*), and object oriented concept lattice *L*_*O*_(*G*, *M*, *I*), respectively. In [27], Yao studied the relations among *L*_*C*_(*G*, *M*, *I*), *L*_*P*_(*G*, *M*, *I*), and *L*_*O*_(*G*, *M*, *I*) and proved these three different lattices are isomorphic. Namely, *L*_*C*_(*G*, *M*, *I*)≅*L*_*P*_(*G*, *M*, *I*)≅*L*_*O*_(*G*, *M*, *I*).

Zhang et al. [17] have ever given detailed approach to find the reduction of a formal context which can keep the structure of *L*(*G*, *M*, *I*). That is, if there exists an attribute subset *D*⊆*M* such that *L*(*G*, *D*, *I*_*D*_)≅*L*(*G*, *M*, *I*), then *D* is called a consistent set of (*G*, *M*, *I*). And, further, if, for all *d* ∈ *D*, *L*(*G*, *D* − *d*, *I*_*D*−*d*_)≇*L*(*G*, *M*, *I*), then *D* is called a reduct of (*G*, *M*, *I*), where *I*_*D*_ = *I*∩(*G* × *D*). According to this idea, the attributes are classified into three types: core attribute, relatively necessary attribute, and absolutely unnecessary attribute.

In this paper, for these four different lattices, we still study attribute reduction based on keeping structures of the lattices. Analogously, the attributes are classified into core attribute, relative necessary attribute, and absolutely unnecessary attribute. To simplify, their attribute reducts are denote by *D*_*i*_. The set of core attributes is *C*_*i*_; that is, *C*_*i*_ = ∩_*j*∈*τ*_ *D*_*ij*_; the set of relatively necessary attributes is *K*_*i*_; that is, *K*_*i*_ = ∪_*j*∈*τ*_ *D*_*ij*_ − ∩_*j*∈*τ*_ *D*_*ij*_; and the set of absolutely unnecessary attributes is *I*_*i*_; that is, *I*_*i*_ = *M* − ∪_*j*∈*τ*_ *D*_*ij*_, where *τ* is an index set, *i* ∈ {*f*, *c*, *p*, *o*}, which represents *L*(*G*, *M*, *I*), *L*_*C*_(*G*, *M*, *I*), *L*_*P*_(*G*, *M*, *I*), and *L*_*O*_(*G*, *M*, *I*), respectively.

An example is given in the following to show the above definitions.

**Example 2 .**

Table 1 is a formal context (*G*, *M*, *I*). *G* = {1,2, 3,4} is an object set and *M* = {*a*, *b*, *c*, *d*, *e*, *f*} is an attribute set.  Table 2 is its complementary context (*G*, *M*, *I* ^*c*^).

**Figure 1:**
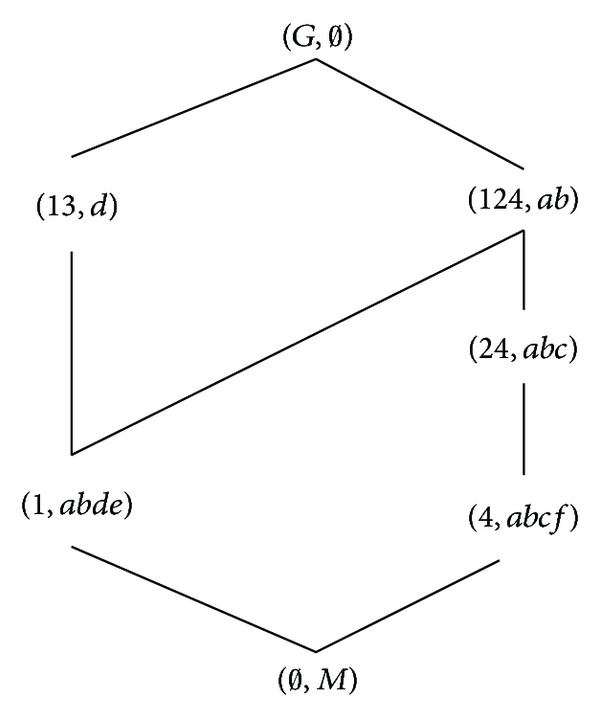
*L*(*G*, *M*, *I*).

**Figure 2:**
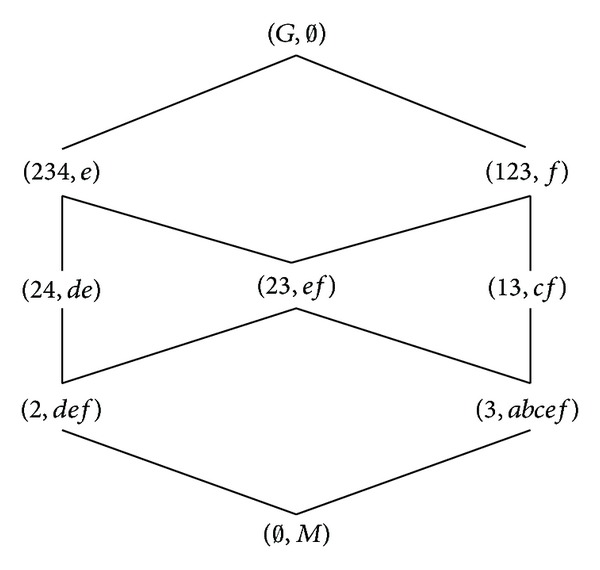
*L*_*C*_(*G*, *M*, *I*).

**Figure 3:**
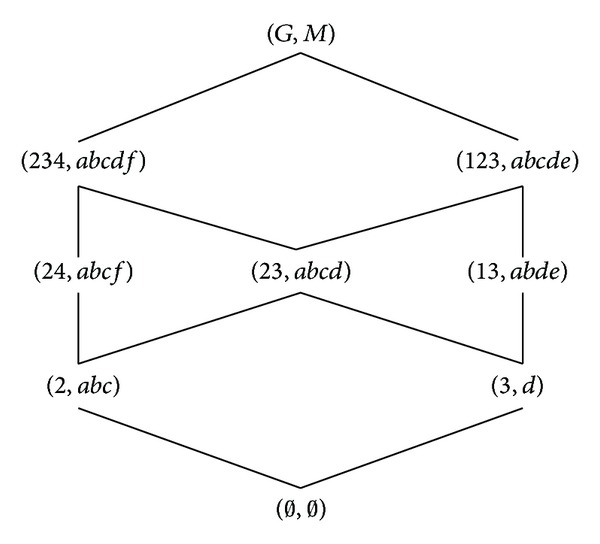
*L*_*P*_(*G*, *M*, *I*).

**Figure 4:**
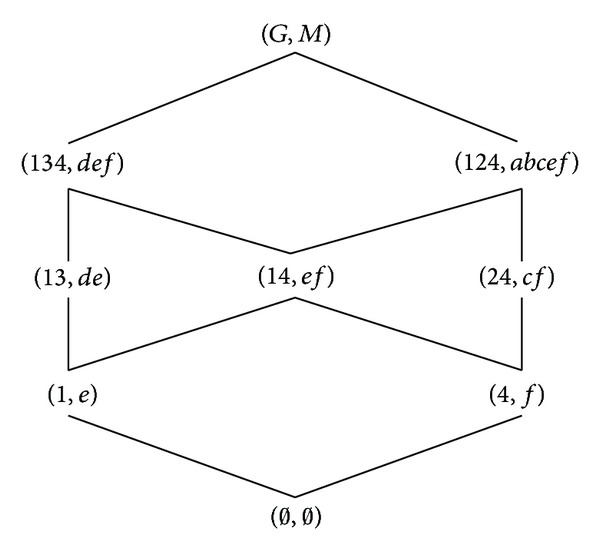
*L*_*O*_(*G*, *M*, *I*).

According to the definitions of formal concept, property oriented concept, and object oriented concept, we can obtain the corresponding concept lattices. The concept lattice *L*(*G*, *M*, *I*) and complementary context concept lattice *L*_*C*_(*G*, *M*, *I*) are shown in Figures 1 and 2. The property oriented concept lattice *L*_*P*_(*G*, *M*, *I*) and the object oriented concept lattice *L*_*O*_(*G*, *M*, *I*) are shown in Figures 3 and 4, respectively, in which every set is denoted directly by listing its elements except *G*, *M*, and *∅*.

For *L*(*G*, *M*, *I*), *C*_*f*_ = {*c*, *d*, *f*}, *K*_*f*_ = {*a*, *b*}, *I*_*f*_ = {*e*}, *D*_*f*1_ = {*a*, *c*, *d*, *f*}, and *D*_*f*2_ = {*b*, *c*, *d*, *f*}.

For *L*_*C*_(*G*, *M*, *I*), *C*_*c*_ = {*c*, *d*, *e*, *f*}, *K*_*c*_ = *∅*, *I*_*c*_ = {*a*, *b*}, and *D*_*c*_ = {*c*, *d*, *e*, *f*}.

For *L*_*P*_(*G*, *M*, *I*), *C*_*p*_ = {*c*, *d*, *e*, *f*}, *K*_*p*_ = *∅*, *I*_*p*_ = {*a*, *b*}, and *D*_*p*_ = {*c*, *d*, *e*, *f*}.

For *L*_*O*_(*G*, *M*, *I*), *C*_*o*_ = {*c*, *d*, *e*, *f*}, *K*_*o*_ = *∅*, *I*_*o*_ = {*a*, *b*}, and *D*_*o*_ = {*c*, *d*, *e*, *f*}.

In Example 2, we noticed that if we remove *a* or *b* from *M*, the structures of four different lattices of the formal context will not be changed. That is, if *m*_1_′ = *m*_2_′ for any *m*_1_, *m*_2_ ∈ *M*, then *m*_1_, *m*_2_ ∉ *C*_*i*_ and *m*_1_, *m*_2_ ∈ *K*_*i*_ or *m*_1_, *m*_2_ ∈ *I*_*i*_.

In order to clarify the situation, we presuppose that the formal context we study in this paper does not have the same column. Here, we delete attribute *b* from Tables 1 and 2. For convenience, we still use *M* as attribute set. But *M* = {*a*, *c*, *d*, *e*, *f*}.

## 3. Attribute Reduction Based on Property Pictorial Diagram

In this section, we mainly propose a method to find attribute reducts of four different lattices based on the property pictorial diagram of a formal context.

### 3.1. Attribute Reduction of *L*(*G*, *M*, *I*)

In the following, we first give the definition of property pictorial diagram.

**Definition 3 .**

Let (*G*, *M*, *I*) be a formal context, *H*_*m*_ = {(*m*′, *m*)∣*m* ∈ *M*}. For any *m*_*s*_, *m*_*t*_ ∈ *M*, if *m*_*s*_′⊆*m*_*t*_′, then one denotes (*m*_*s*_′, *m*_*s*_)≤(*m*_*t*_′, *m*_*t*_). And (*H*_*m*_, ≤) is called the property pictorial diagram of (*G*, *M*, *I*).

In fact, the Hasse diagram (*H*_*m*_, ≤) gives another expression of (*G*, *M*, *I*). The diagrammatic approach to formal context obtains the relations among attribute extents easily.

**Definition 4 (see [1]).**

*a* is called a lower neighbor of  *b*, if *a* < *b* and there is no element of *c* fulfilling *a* < *c* < *b*. In this case, *b* is an upper neighbor of *a*, and one writes *a*≺*b*.

Based on this definition, we can easily obtain upper neighbors and lower neighbors of each element (*m*′, *m*) in *H*_*m*_. For any *m* ∈ *M*, denote *U*_*m*_ = {*m*_*t*_ ∈ *M*∣(*m*′, *m*)≺(*m*_*t*_′, *m*_*t*_)} and *L*_*m*_ = {*m*_*s*_ ∈ *M*∣(*m*_*s*_′, *m*_*s*_)≺(*m*′, *m*)}, where *s* ∈ *S*, *t* ∈ *T* (*S* and *T* are index sets).

**Example 5 (continue with Example 2).**

**Figure 5:**
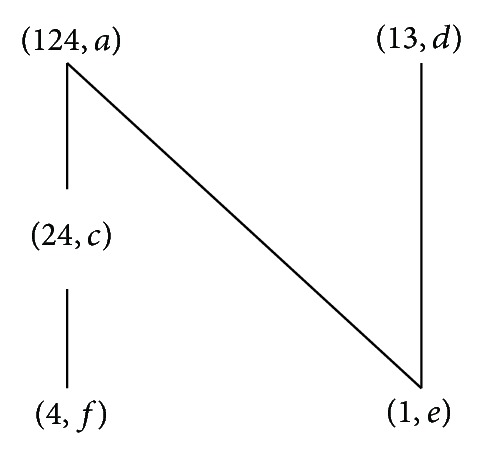
(*H*_*m*_, ≤) of (*G*, *M*, *I*) in Table 1.

Consider the formal context in Table 1; we have *a*′ = 124, *c*′ = 24, *d*′ = 13, *e*′ = 1, and *f*′ = 4. According to Definition 3, we have *H*_*m*_ = {(124, *a*), (24, *c*), (13, *d*), (1, *e*), (4, *f*)}, and the property pictorial diagram is shown in Figure 5. Thus, we have

 
*U*_*a*_ = *∅* , *U*_*c*_ = {*a*}, *U*_*d*_ = *∅*, *U*_*e*_ = {*a*, *d*}, *U*_*f*_ = {*c*},
 
*L*_*a*_ = {*c*, *e*}, *L*_*c*_ = {*f*}, *L*_*d*_ = {*e*}, *L*_*e*_ = *∅*, *L*_*f*_ = *∅*.

It is easy to see that the maximal elements of *H*_*m*_ have no upper neighbor and the minimal elements of *H*_*m*_ have no lower neighbor. We denote the set of maximal and minimal elements of *H*_*m*_ by *Max*⁡(*H*_*m*_) and *Min*⁡(*H*_*m*_), respectively.

In [1], the arrow relation ↗ on the (*G*, *M*, *I*) was defined as follows: *g*↗*m* : ⇔¬(*gIm*) and if *m*′⊆*n*′ and *m*′ ≠ *n*′, then *gIn*, where *g* ∈ *G*, *m*, *n* ∈ *M*. In the following, we will give a new method to obtain the arrow relation ↗ based on property pictorial diagram (*H*_*m*_, ≤).

**Theorem 6 .**

Let (*G*, *M*, *I*) be a formal context and let (*H*_*m*_, ≤) be its property pictorial diagram. The following statements hold.

1. If (*m*′, *m*) ∈ *Max*⁡(*H*_*m*_), then *g* ∈ *G* − *m*′⇔*g*↗*m*.
2. If (*m*′, *m*) ∉ *Max*⁡(*H*_*m*_), then *g* ∈ ⋂_*t*∈*T*_ *m*_*t*_′ − *m*′⇔*g*↗*m*, where *m*_*t*_ ∈ *U*_*m*_ (*t* ∈ *T*).

**Proof**

(1) Suppose (*m*′, *m*) ∈ *Max*⁡(*H*_*m*_). Thus, there does not exist (*m*_*t*_′, *m*_*t*_) ∈ *H*_*m*_ such that *m*′ ⊂ *m*_*t*_′. And, by *g* ∈ *G* − *m*′, we have ¬(*gIm*). So, from the definition of ↗, we have *g*↗*m*. Hence, for any (*m*′, *m*) ∈ *Max*⁡(*H*_*m*_), *g* ∈ *G* − *m*′⇒*g*↗*m*.

Conversely, because the formal context is canonical, *G* − *m*′ ≠ *∅*. And since (*m*′, *m*) ∈ *Max*⁡(*H*_*m*_) and *g*↗*m*, we have *g* ∈ *G* − *m*′ from the definition of ↗. Thus, for any (*m*′, *m*) ∈ *Max*⁡(*H*_*m*_), *g*↗*m*⇒*g* ∈ *G* − *m*′.

(2) Suppose (*m*′, *m*) ∉ *Max*⁡(*H*_*m*_). According to the definition of maximal elements, there exists (*m*_*t*_′, *m*_*t*_) ∈ *H*_*m*_ such that *m*′ ⊂ *m*_*t*_′. And, by *g* ∈ ⋂_*t*∈*T*_ *m*_*t*_′ − *m*′, we have *g* ∉ *m*′ and *g* ∈ *m*_*t*_′; that is ¬(*gIm*), *gIm*_*t*_. So we have *g*↗*m* from the definition of ↗.

Since (*m*′, *m*) ∉ *Max*⁡(*H*_*m*_), there exist some *m*_*t*_ ∈ *M* such that *m*′ ⊂ *m*_*t*_′. And by *g*↗*m*, we have *g* ∉ *m*′ and *g* ∈ *m*_*t*_′; that is, ¬(*gIm*), *gIm*_*t*_. So *g* ∈ ⋂_*t*∈*T*_ *m*_*t*_′ − *m*′.

**Example 7 (continue with Example 2).**

From Theorem 6, we can obtain the arrow relation ↗ of Table 1 based on (*H*_*m*_, ≤); it is illustrated in Table 3.

Here, we recall an important definition as follows.

**Definition 8 (see [28]).**

Let *L* be a lattice. An element *x* ∈ *L* is meet-irreducible if

1. *x* ≠ 1 (in case *L* has a unit),
2. *x* = *a*∧*b* implies *x* = *a* or *x* = *b* for all *a*, *b* ∈ *L*.

We denote the set of meet-irreducible elements of *L*(*G*, *M*, *I*) by *M*(*L*).

Based on the arrow relations ↗, Ganter and Wille gave the method to judge whether an attribute concept is a meet-irreducible element of *L*(*G*, *M*, *I*).

**Lemma 9 (see [1]).**

The following statements hold for every context: (*m*′, *m* ^′∗^) ∈ *M*(*L*)⇔ there is a *g* ∈ *G* with *g*↗*m*.

According to the properties of meet-irreducible elements of concept lattices, Wang and Ma [20] gave the judgement method of absolutely unnecessary attributes.

**Lemma 10 (see [20]).**

If (*G*, *M*, *I*) is a context, for any *m* ∈ *M*, one has

(6) $$\begin{array}{c} m\in I_{f}⟺\left(m^{\prime },m^{\prime *}\right)\notin M\left(L\right). \end{array}$$

Combining these two lemmas, we have the following result.

**Theorem 11 .**

Let (*G*, *M*, *I*) be a formal context and let (*H*_*m*_, ≤) be its property pictorial diagram. For any *m* ∈ *M*, one has *m* ∈ *I*_*f*_⇔(*m*′, *m*) ∉ *Max*⁡(*H*_*m*_) and ⋂_*t*∈*T*_ *m*_*t*_′ − *m*′ = *∅*, where *m*_*t*_ ∈ *U*_*m*_ (*t* ∈ *T*).

**Proof**

From Lemmas 9 and 10, it is easy to see that (*m*′, *m* ^′∗^) ∉ *M*(*L*)⇔ there does not exist *g* ∈ *G* with *g*↗*m*.

According to Theorem 6, we obtain that there does not exist *g* ∈ *G* with *g*↗*m*⇔(*m*′, *m*) ∉ *Max*⁡(*H*_*m*_) and ⋂_*t*∈*T*_ *m*_*t*_′ − *m*′ = *∅*. Then, this theorem is proved.

Theorem 11 shows that if |*U*_*m*_| ≤ 1 for *m* ∈ *M*, then *m* ∉ *I*_*f*_.

Because the formal contexts we study do not have the same column, that is, there is no relatively necessary attribute, we can get the following statement.

**Theorem 12 .**

Let (*G*, *M*, *I*) be a formal context. One has *D*_*f*_ = *M*∖*I*_*f*_.

By this theorem, we can obtain an attribute reduct of *L*(*G*, *M*, *I*). The steps are as follows.

1. Compute *m*′ for all *m* ∈ *M*.
2. Draw the property pictorial diagram (*H*_*m*_, ≤).
3. Find *U*_*m*_. If |*U*_*m*_| ≥ 2 and *m*′ = ⋂_*m*_*t*_∈*U*_*m*__ *m*_*t*_′, then *m* ∈ *I*_*f*_.
4. Obtain an attribute reduct *D*_*f*_ = *M*∖*I*_*f*_.

**Example 13 (continue with Example 5).**

Example 5 told us that |*U*_*a*_| = 0, |*U*_*c*_| = 1, |*U*_*d*_| = 0, |*U*_*e*_| = 2, and |*U*_*f*_| = 1. From Theorem 11, we only need to check attribute *e*. Because *U*_*e*_ = {*a*, *d*} and *a*′∩*d*′ = *e*′, we have *e* ∈ *I*_*f*_. Thus, *D*_*f*_ = {*a*, *c*, *d*, *f*}. The result is consistent with Example 2.

### 3.2. Attribute Reduction of *L*_*C*_(*G*, *M*, *I*), *L*_*O*_(*G*, *M*, *I*), and *L*_*P*_(*G*, *M*, *I*)

For a formal context, its complementary context is unique and *L*_*C*_(*G*, *M*, *I*)≅*L*_*O*_(*G*, *M*, *I*)≅*L*_*P*_(*G*, *M*, *I*). Therefore, we will discuss the attribute reduction of these three different lattices based on the property pictorial diagram of original formal context.

For the complementary context (*G*, *M*, *I* ^*c*^) of (*G*, *M*, *I*), we denote its property pictorial diagram by (*H*_*m*_^*C*^, ≤).

**Theorem 14 .**

Let (*G*, *M*, *I*) be a formal context and let (*H*_*m*_, ≤) be its property pictorial diagram. For any (*m*′, *m*) ∈ *H*_*m*_, one has

1. (~*m*′, *m*) ∈ *H*_*m*_^*C*^,
2. (*m*′, *m*)≺(*m*_*t*_′, *m*_*t*_)⇔(~*m*_*t*_′, *m*_*t*_)≺(~*m*′, *m*) (*t* ∈ *T*),
3. *Max*⁡(*H*_*m*_^*C*^) = {(~*m*′, *m*)(*m*′, *m*) ∈ *Min*⁡(*H*_*m*_)},
4. *H*_*m*_≅*H*_*m*_^*C*^.

**Proof**

1. From the definition of complementary context, we know that *m* ^′*c*^ = ~*m*′. Thus (~*m*′, *m*) = (*m* ^′*c*^, *m*). Hence, we have (~*m*′, *m*) ∈ *H*_*m*_^*C*^ by Definition 4.
2. Consider (*m*′, *m*)≺(*m*_*t*_′, *m*_*t*_)⇔*m*′ ⊂ *m*_*t*_′⇔~*m*_*t*_′ ⊂ ~*m*′⇔(~*m*_*t*_′, *m*_*t*_)≺(~*m*′, *m*).
3. It is easy to be obtained from (2).
4. It can be proved by (1) and (2).

For the complementary context (*G*, *M*, *I* ^*c*^), we have the following result from Theorems 6 and 14.

**Theorem 15 .**

Let (*G*, *M*, *I*) be a formal context and let (*H*_*m*_, ≤) be its property pictorial diagram. The following statements hold.

1. If (*m*′, *m*) ∈ *Min*⁡(*H*_*m*_), then *g* ∈ *m*′⇔*g*↗*m* in (*G*, *M*, *I* ^*c*^).
2. If (*m*′, *m*) ∉ *Min*⁡(*H*_*m*_), then *g* ∈ *m*′ − ⋃_*s*∈*S*_ *m*_*s*_′⇔*g*↗*m* in (*G*, *M*, *I* ^*c*^), where *m*_*s*_ ∈ *L*_*m*_ (*s* ∈ *S*).

**Example 16 (continue with Example 2).**

**Figure 6:**
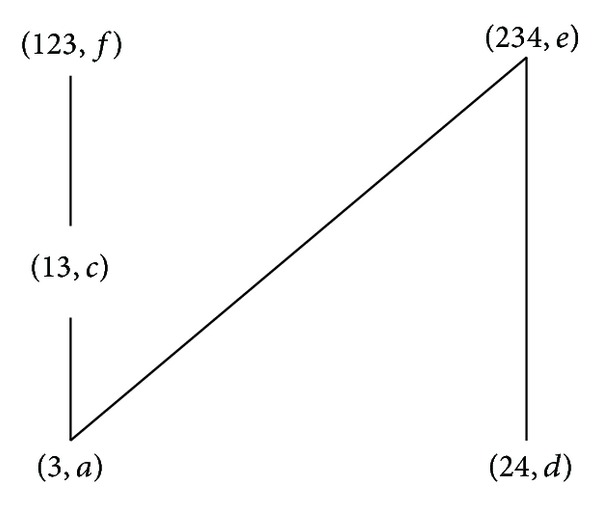
(*H*_*m*_^*C*^, ≤) of (*G*, *M*, *I* ^*c*^) in Table 2.

Consider the formal context in Table 2. According to Definition 3, we have *H*_*m*_^*C*^ = {(3, *a*), (13, *c*), (24, *d*), (234, *e*), (123, *f*)} and the property pictorial diagram is in Figure 6.

It is easy to verify Theorem 14 by Figures 5 and 6. By Theorem 15, the arrow relation ↗ of Table 2 can be obtained as Table 4.

Combining Lemmas 9 and 10, we have the following conclusion similar to Theorem 11.

**Theorem 17 .**

Let (*G*, *M*, *I*) be a formal context and let (*H*_*m*_, ≤) be its property pictorial diagram. For any *m* ∈ *M*, one has *m* ∈ *I*_*c*_⇔(*m*′, *m*) ∉ *Min*⁡(*H*_*m*_) and *m*′ − ⋃_*s*∈*S*_ *m*_*s*_′ = *∅*, where *m*_*s*_ ∈ *L*_*m*_ (*s* ∈ *S*).

This theorem implies that if |*L*_*m*_| ≤ 1 for *m* ∈ *M*, then *m* ∉ *I*_*c*_.

Similar to Theorem 12, we have the following result.

**Theorem 18 .**

Let (*G*, *M*, *I*) be a formal context. One has *D*_*c*_ = *M*∖*I*_*c*_.

By Theorem 18, we can obtain an attribute reduct of *L*_*C*_(*G*, *M*, *I*).

In [22], Medina studied attribute reduction of object oriented concept lattice and property oriented concept lattice using the relations between these two lattices and complementary context concept lattice in a formal context. The main conclusions are as follows.

**Theorem 19 (see [22]).**

Let (*G*, *M*, *I*) be a formal context. For all *m* ∈ *M*, one has the following:

1. *m* ∈ *I*_*p*_⇔*m* ∈ *I*_*o*_⇔*m* ∈ *I*_*c*_,
2. *m* ∈ *K*_*p*_⇔*m* ∈ *K*_*o*_⇔*m* ∈ *K*_*c*_,
3. *m* ∈ *C*_*p*_⇔*m* ∈ *C*_*o*_⇔*m* ∈ *C*_*c*_,
4. *D*_*c*_ = *D*_*p*_ = *D*_*o*_.

Combing Theorems 18 and 19, the corresponding reduction process is as follows.

1. Compute *m*′ for all *m* ∈ *M*.
2. Draw the property pictorial diagram (*H*_*m*_, ≤).
3. Find *L*_*m*_. If |*L*_*m*_| ≥ 2 and *m*′ = ⋃_*m*_*s*_∈*L*_*m*__ *m*_*s*_′, then *m* ∈ *I*_*c*_.
4. Obtain attribute reducts *D*_*c*_ = *D*_*p*_ = *D*_*o*_ = *M*∖*I*_*c*_.

**Example 20 (continue with Example 5).**

According to Example 5, we obtain |*L*_*a*_| = 2, |*L*_*c*_| = 1, |*L*_*d*_| = 1, |*L*_*e*_| = 1, and |*L*_*f*_| = 1. We only need to check attribute *a*. Because *L*_*a*_ = {*c*, *e*} and *a*′ = *c*′ ∪ *e*′, we have *a* ∈ *I*_*c*_ by Theorem 17. Thereby, *D*_*c*_ = *D*_*p*_ = *D*_*o*_ = {*c*, *d*, *e*, *f*}. These results are consistent with Example 2.

## 4. An Illustrated Example

**Example 1 .**

To illustrate the application of the method proposed by this paper we use the data set of bacterial taxonomy from UCI. The data set contains six species and 16 phenotypic characters.  Table 5 shows the formal context (*G*, *M*, *I*) of the bacterial data set. We denote *G* = {1,2, 3,4, 5,6, 7,8, 9,10,11,12,13,14,15,16,17} and *M* = {*a*, *b*, *c*, *d*, *e*, *f*, *g*, *h*, *i*, *j*, *k*, *l*, *m*, *n*, *o*, *p*}. The species are* Escherichia coli* (1–3),* Salmonella typhi* (4–6),* Klebsiella pneumoniae* (7–11),* Proteus vulgaris* (12–14),* Proteus morganii* (15, 16), and* Serratia marcesens* (17), respectively.

First, compute attribute extents *m*′ for all *m* ∈ *M* as follows:

 
*a*′ = {3,6, 12,13,14},
 
*b*′ = {1,2, 3,4, 5,6, 7,8, 9,10,11,17},
 
*c*′ = {1,4, 5,6, 7,8, 9,10,11,15,17},
 
*d*′ = {1,2, 3,7, 8,9, 10,11,12,13,14,15,16},
 
*e*′ = {2,3, 15,16,17},
 
*f*′ = {7,8, 9,10,11,12,17},
 
*g*′ = {7,8, 9,10,12,14,15},
 
*h*′ = {1,2, 3,7, 8,9, 10,11,17},
 
*i*′ = {7,8, 9,11,17},
 
*j*′ = {7,8, 9,10,11},
 
*k*′ = {17},
 
*l*′ = {12,13,14,15},
 
*m*′ = {8,9, 10,11},
 
*n*′ = {7,9, 10,11},
 
*o*′ = {1,2, 3,4, 6,7, 8,9, 10,11},
 
*p*′ = {1,3, 7,8, 9,10,11}.

**Figure 7:**
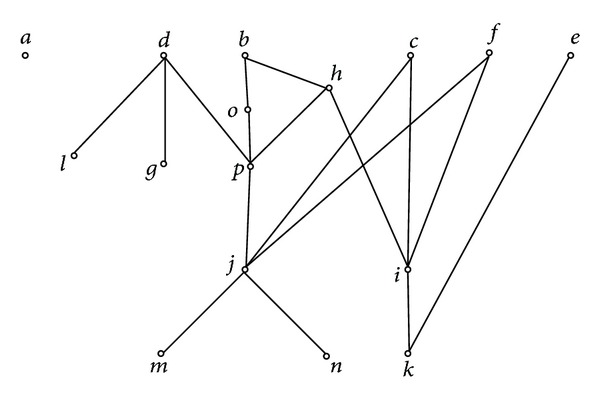
(*H*_*m*_, ≤) of (*G*, *M*, *I*) in Table 5.

Second, draw the property pictorial diagram. Here, for clarification, every element of property pictorial diagram is denoted directly by the corresponding attribute label, which is shown in Figure 7.

Third, for any *m* ∈ *M*, compute *U*_*m*_ and *L*_*m*_ (Table 6).

According to Theorem 11, we only need to examine attributes *i*, *j*, *k*, and *p*. We have the following:

 
*U*_*i*_ = {*c*, *h*, *f*} and *c*′∩*h*′∩*f*′ − *i*′ = {10}, *i* ∉ *I*_*f*_,
 
*U*_*j*_ = {*c*, *f*, *p*} and *c*′∩*f*′∩*p*′ − *j*′ = *∅*, *j* ∈ *I*_*f*_,
 
*U*_*k*_ = {*e*, *i*} and *e*′∩*i*′ − *k*′ = *∅*, *k* ∈ *I*_*f*_,
 
*U*_*p*_ = {*d*, *h*, *o*} and *d*′∩*h*′∩*o*′ − *p*′ = {2}, *p* ∉ *I*_*f*_.

According to Theorem 17, we only need to examine attributes *b*, *c*, *d*, *f*, *h*, and *j*. We have the following:

 
*L*_*b*_ = {*h*, *o*} and *b*′ − *h*′ ∪ *o*′ = {5}, *b* ∉ *I*_*c*_,
 
*L*_*c*_ = {*i*, *j*} and *c*′ − *i*′ ∪ *j*′ = {1,4, 5,6, 15}, *c* ∉ *I*_*c*_,
 
*L*_*d*_ = {*g*, *l*, *p*} and *d*′ − *j*′ ∪ *l*′ ∪ *p*′ = {2,16}, *d* ∉ *I*_*c*_,
 
*L*_*f*_ = {*i*, *j*} and *f*′ − *i*′ ∪ *j*′ = {12}, *f* ∉ *I*_*c*_,
 
*L*_*h*_ = {*i*, *p*} and *h*′ − *i*′ ∪ *p*′ = {2}, *h* ∉ *I*_*c*_,
 
*L*_*j*_ = {*m*, *n*} and *j*′ − *m*′ ∪ *n*′ = *∅*, *j* ∈ *I*_*c*_.

Fourth, we obtain *D*_*f*_ = {*a*, *b*, *c*, *d*, *e*, *f*, *g*, *h*, *i*, *l*, *m*, *n*, *o*, *p*}, *D*_*c*_ = *D*_*o*_ = *D*_*p*_ = {*a*, *b*, *c*, *d*, *e*, *f*, *g*, *h*, *i*, *k*, *l*, *m*, *n*, *o*, *p*}.

## 5. Conclusion

Attribute reduction to keep the lattice structure is an important issue in FCA. On the basis of equivalent relation, the paper presents a new expression for a formal context, which is named property pictorial diagram. According to the property pictorial diagram of original formal context, we propose a method to obtain attribute reducts of four different lattices using the interconnection between arrow relation ↗, meet-irreducible elements, and absolutely unnecessary attributes. Based on the method in this paper, we can study other types of attribute reduction.

## Figures and Tables

**Table 1:** A formal context (*G*, *M*, *I*).

*G*	*a*	*b*	*c*	*d*	*e*	*f*
1	×	×		×	×	
2	×	×	×			
3				×		
4	×	×	×			×

**Table 2:** A formal context (*G*, *M*, *I* ^*c*^).

*G*	*a*	*b*	*c*	*d*	*e*	*f*
1			×			×
2				×	×	×
3	×	×	×		×	×
4				×	×	

**Table 3:** The arrow relation ↗ of (*G*, *M*, *I*).

*G*	*a*	*c*	*d*	*e*	*f*
1	×	↗	×	×	
2	×	×	↗		↗
3	↗		×		
4	×	×	↗		×

**Table 4:** The arrow relation ↗ of (*G*, *M*, *I* ^*c*^).

*G*	*a*	*c*	*d*	*e*	*f*
1		×		↗	×
2		↗	×	×	×
3	×	×	↗	×	×
4			×	×	↗

**Table 5:** Original formal context (*G*, *M*, *I*) from the bacterial data set.

*G*	*a*	*b*	*c*	*d*	*e*	*f*	*g*	*h*	*i*	*j*	*k*	*l*	*m*	*n*	*o*	*p*
1		×	×	×				×							×	×
2		×		×	×			×							×	
3	×	×		×	×			×							×	×
4		×	×												×	
5		×	×													
6	×	×	×												×	
7		×	×	×		×	×	×	×	×				×	×	×
8		×	×	×		×	×	×	×	×			×		×	×
9		×	×	×		×	×	×	×	×			×	×	×	×
10		×	×	×		×	×	×		×			×	×	×	×
11		×	×	×		×		×	×	×			×	×	×	×
12	×			×		×	×					×				
13	×			×								×				
14	×			×			×					×				
15			×	×	×		×					×				
16				×	×							×				
17		×	×		×	×		×	×		×					

**Table 6:** 

*U*_*a*_ = *∅*	*U*_*b*_ = ∅
*L*_*a*_ = ∅	*L*_*b*_ = {*o*, *h*}

*U*_*c*_ = ∅	*U*_*d*_ = ∅
*L*_*c*_ = {*i*, *j*}	*L*_*d*_ = {*g*, *l*, *p*}

*U*_*e*_ = ∅	*U*_*f*_ = ∅
*L*_*e*_ = {*k*}	*L*_*f*_ = {*i*, *j*}

*U*_*g*_ = {*d*}	*U*_*h*_ = {*b*}
*L*_*g*_ = ∅	*L*_*h*_ = {*i*, *p*}

*U*_*i*_ = {*c*, *h*, *f*}	*U*_*j*_ = {*c*, *f*, *p*}
*L*_*i*_ = {*k*}	*L*_*j*_ = {*m*, *n*}

*U*_*k*_ = {*e*, *i*}	*U*_*l*_ = {*d*}
*L*_*k*_ = ∅	*L*_*l*_ = ∅

*U*_*m*_ = {*j*}	*U*_*n*_ = {*j*}
*L*_*m*_ = ∅	*L*_*n*_ = ∅

*U*_*o*_ = {*b*}	*U*_*p*_ = {*d*, *h*, *o*}
*L*_*o*_ = {*p*}	*L*_*p*_ = {*j*}
